# Liquid Crystals: A Novel Approach for Cancer Detection and Treatment

**DOI:** 10.3390/cancers10110462

**Published:** 2018-11-21

**Authors:** Jayalakshmi Vallamkondu, Edwin Bernard Corgiat, Gollapelli Buchaiah, Ramesh Kandimalla, P. Hemachandra Reddy

**Affiliations:** 1 Department of Physics, NIT Warangal, Telangana 506004, India; buchaiahg@gmail.com; 2 Centre for Advanced Materials, NIT Warangal, Telangana 506004, India; 3 Department of Cellular Biology, Emory University School of Medicine, Atlanta, GA 30322, USA; edwin.bernard.corgiat@emory.edu; 4 Garrison Institute on Aging, Texas Tech University Health Sciences Center, 3601 4th Street, MS 9424, Lubbock, TX 79430, USA; ramesh.kandimalla@ttuhsc.edu; 5 Neurology Department, Texas Tech University Health Sciences Center, 3601 4th Street, MS 9424, Lubbock, TX 79430, USA; 6 Pharmacology and Neuroscience Department, Texas Tech University Health Sciences Center, 3601 4th Street, MS 9424, Lubbock, TX 79430, USA; 7 Garrison Institute on Aging, South West Campus, Texas Tech University Health Sciences Center, 6630 S. Quaker Suite E, MS 7495, Lubbock, TX 79413, USA; 8 Cell Biology and Biochemistry Department, Texas Tech University Health Sciences Center, 3601 4th Street, MS 9424, Lubbock, TX 79430, USA; 9 Speech, Language and Hearing Sciences Department, Texas Tech University Health Sciences Center, 3601 4th Street, MS 9424, Lubbock, TX 79430, USA; 10 Department of Public Health, Graduate School of Biomedical Sciences, 3601 4th Street, MS 9424, Lubbock, TX 79430, USA

**Keywords:** cancers, liquid crystals, biomarker, biosensor, antitumor drug

## Abstract

Liquid crystals are defined as the fourth state of matter forming between solid and liquid states. Earlier the applications of liquid crystals were confined to electronic instruments, but recent research findings suggest multiple applications of liquid crystals in biology and medicine. Here, the purpose of this review article is to discuss the potential biological impacts of liquid crystals in the diagnosis and prognosis of cancer along with the risk assessment. In this review, we also discussed the recent advances of liquid crystals in cancer biomarker detection and treatment in multiple cell line models. Cases reviewed here will demonstrate that cancer diagnostics based on the multidisciplinary technology and intriguingly utilization of liquid crystals may become an alternative to regular cancer detection methodologies. Additionally, we discussed the formidable challenges and problems in applying liquid crystal technologies. Solving these problems will require great effort and the way forward is through the multidisciplinary collaboration of physicists, biologists, chemists, material-scientists, clinicians, and engineers. The triumphant outcome of these liquid crystals and their applications in cancer research would be convenient testing for the detection of cancer and may result in treating the cancer patients non-invasively.

## 1. Introduction

**Figure 1:**
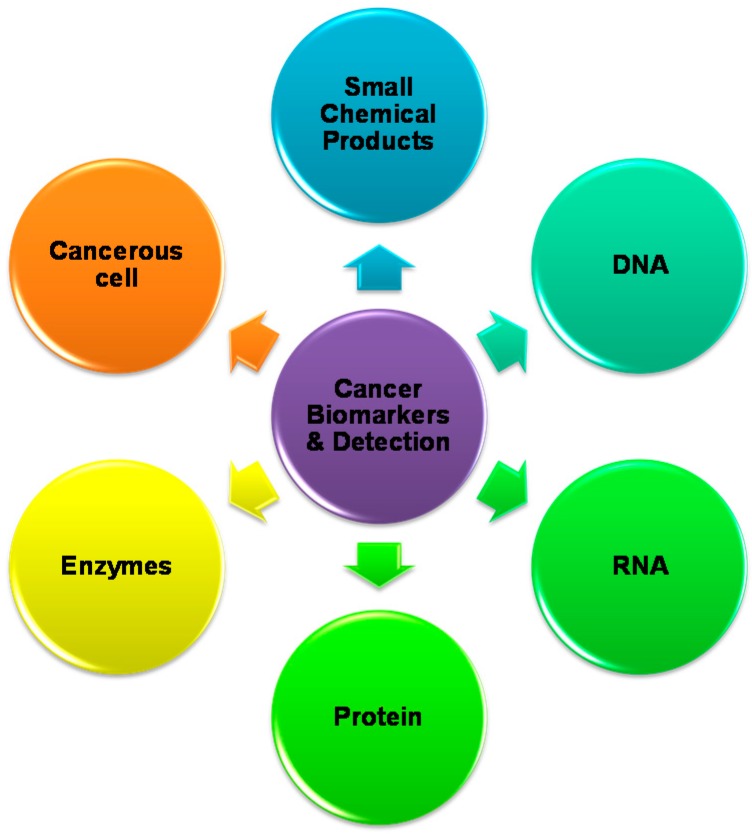
Biological nature and types of cancer bio-markers.

Cancer is defined as uncontrolled division of abnormal cells, so early detection of cancer can significantly improve patient treatment outcome and saves lives. In the past decade, there have been considerable improvements in the way human tumors are treated, largely due to increased understanding of cancer at the molecular level. Researchers around the globe have spent lot of time, hard work, and money to develop simple techniques for early detection of the cancer disease. As per the National Cancer Institute, a biomarker is characterized as “an organic particle found in tissues, other body liquids, or blood that can be impartially estimated and assessed as an indication of an ordinary or irregular process, or of a condition or illness,” and can be utilized, for instance, to separate a persistent growth from one without the infection [1]. Disease can form from adjustments to various variables, including germline or substantial transformations, transcriptional changes, and posttranslational alterations. There are a large number of biomarkers including proteins (e.g., a compound or receptor), nucleic acids (e.g., a microRNA or other non-coding RNA), antibodies, peptides, and more. A biomarker can likewise be a group of changes, for example, quality articulation, proteomic, and metabolomics marks. Growth biomarkers, including DNA, mRNA, catalysts, cell surface receptors, interpretation factors, antibodies, peptides, non-coding RNA, and metabolites, are available in serum and tumor tissues (Figure 1) [2]. These factors become more pronounced throughout cancer progression. Discovery of cancer disease biomarkers is an imperative measure to assess in malignancy counteractive action and early screening, and also in tumor assessment and forecast. Thus, clinical tests of different growth biomarkers are screened for early improvement, headway, or conceivable repeat of disease. Furthermore, these measures can serve to evaluate a patient’s reaction to the treatment of malignancy, hence supplementing the physical examination. The most common cancer treatments for cancer patients are radiation, chemotherapy, and surgery. The chemotherapy approaches can manage uncontrolled advancement of irregular malignancy cells, and it is regularly joined with medical procedures or radiation treatment. The chemotherapeutic medications can be anthracyclines, alkylating chemical agents, plant alkaloids, antimetabolites, topoisomerase inhibitors, monoclonal antibodies, or other antitumor molecules [3,4,5,6,7]. There are numerous studies that aim to detect cancer biomarkers and to develop antitumor drugs that have molecular targets [8,9]. Therefore, the advancement of more compelling techniques for immuno-detection of cancer biomarkers and expanded viability of chemotherapy drugs is fundamental for the treatment of cancer.

**Figure 2:**
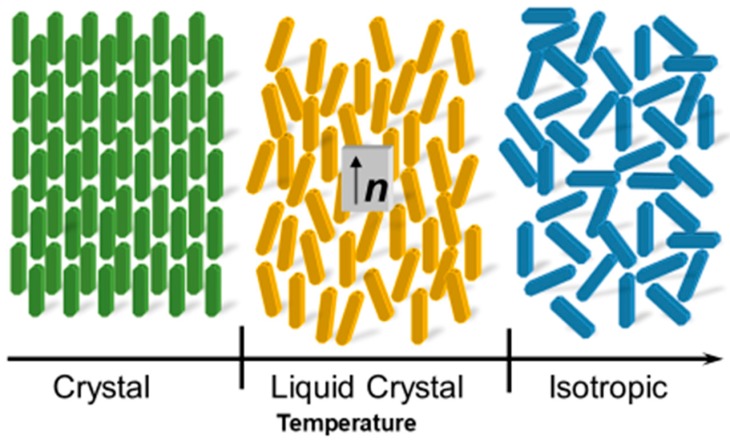
Schematic representation of arrangement of molecules in the crystal, liquid, crystal and isotropic phase with increase of temperature.

**Figure 3:**
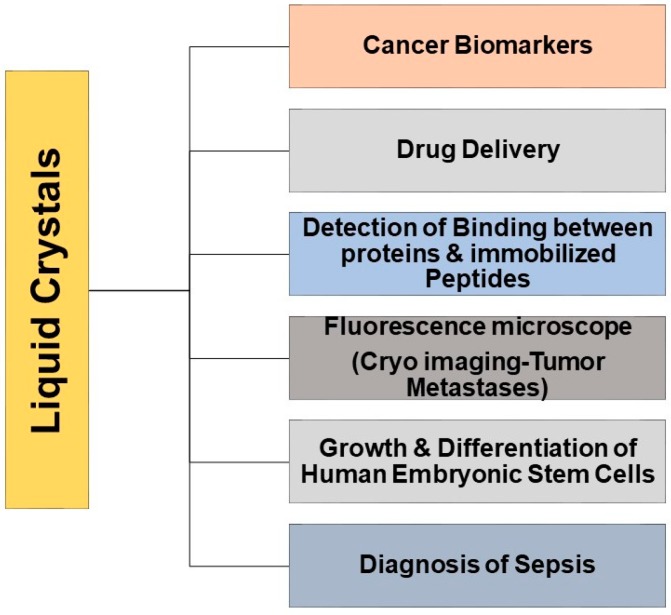
Biological applications of liquid crystals in medicine.

Liquid crystal is a kind of matter existing between the crystalline and liquid phases, and has distinctive features including the partial and or complete loss of the constituent anisotropic molecules positional order in state of matter specific way (Figure 2) [10]. Liquid crystals (LCs) are technologically vital electro-optical materials as they have numerous remarkable and helpful optical and physical properties. [11,12,13,14,15,16]. In general LCs are widely used in television screens as LCD (liquid crystal display) display, in display media, and in personal computers [15]. Usually, these LCs can be produced from numerous precursors including a flexible chain, a rigid aromatic core, and hydrophobic and hydrophilic units. Furthermore, different organic cell structures, for example, cell layers, comprise of amphiphilic phospholipids. In this way, it is conceivable that LCs or their precursors, liquid crystal related compounds (LCRCs), can interact with biological cell structures. There has been an increasing thrust to uncover the natural and pharmacological impacts of these crystal molecules [17,18,19]. Recently, applications of LCs in biological sensing and drug delivery have received increased attention for use in sensing or treatment of analgesics, anti-cancer, liver diseases, anti-asthmatic, nanoparticles formulations, and more [20,21]. These LCs worked based on the biosensor activity due to the birefringent properties of the anisotropic molecules. This birefringent orientation during the process of the biological event and or in the presence of allows a change in optical appearance of the LC utilized [22]. Interestingly, phospholipids play a crucial role in changing the configuration of nematic liquid crystals from planar to homeotropic and it is due to the assembly of phospholipids between the nematic LC 4-Cyano-4′-pentylbiphenyl (5CB) and aqueous phase. This allows to change the optical texture of 5CB from dark to bright when observed under a microscope (crossed polarizers) called a polarized optical microscope (POM) [23]. Similarly, the LCs have been used to detect the association and nature of the bond between immobilized peptides and proteins, to recognize the orientation of immobilized proteins [24,25,26], and to monitor real-time enzymatic reactions [27,28]. In addition, investigators also found the role of LCs especially thermotropic LCs in stem cell research and they were used in the growth of embryonic stem cells and embryogenesis. The importance of LCs were exploited in the reorganization of the extracellular matrix and also during growth and differentiation as shown in neurodegenerative diseases such Alzheimer’s disease, Gaucher’s disease (lysosomal storage disorder), steatohepatitis mouse models, and atherosclerosis [29]. Moreover, in addition to the above applications of LCs, few investigators used “the dependent properties of LCs” to differentiate the lipid bilayer enveloped viruses and non-enveloped viruses [30], in the diagnosis of sepsis [31] (Figure 3). These various biological functions, combined with the advantages of being rapid, label-free, and low-cost, exhibit the potential role of LC based bio-sensing in diagnostics and clinical applications [32]. Many investigators have employed liquid crystals in detection of cancer biomarkers but to date, none were reviewed about the role of liquid crystals in cancer biology and biomarkers in the literature to the best of our knowledge. Even though there has been little discussion about LCs in the detection of cancer biomarkers, rapid progress is being made in the LC field. In recent years, a lot of research work has been carried out leading to a few new technical concepts and identification tools, which alone merit a review.

In this review paper, we discuss the ongoing progress of liquid crystals in cancer biomarker detection and treatment. We additionally endeavor to give an exhaustive review covering the current difficulties and most recent improvements to accomplish high sensitivity and selectivity for the discovery of different cancer biomarkers. It is believed that our review paper may motivate and inspire researcher’s interests about the liquid crystals applications in multiple disciplines and also enhance exciting developments in this field which holds much promise for benefitting of human health.

## 2. Liquid Crystal Based Detection of Cancer

### 2.1. Detection of the Cancer Biomarker CA125: Label-Free Immunodetection

Immunodetection is a vital procedure in the detection of cancer detection and its diagnosis. The biomarkers for the cancer are “specific molecules” or “proteins” released by cancer cells. These kinds of biomarkers are present in blood and urine in high quantities and are eventually helpful in detecting and evaluating the stages of cancer, progression and patient response towards the treatment. Traditional methods such as immunohistochemistry, flow cytometry, and polymerase chain reaction (PCR) [33,34,35] are very sensitive and precise in detecting various cancer cells but they are time-consuming and expensive. The most widely used methods currently in cancer screening are ELISA (Enzyme linked immunosorbent assay) based assays, where the strength of the signal is based on the amount of light (chemiluminescence) released from labeled molecules and the signal strength directly analyzed for the quantification of the level of biomarkers in the diagnosis of cancer. Due to the length and complexity of these assays, novel methods have been invented to detect immunocomplexes more accurately and with greater sensitivity. These include optical immunosensor, protein coated chips, electrochemical and nanoparticles based immunological assays [36,37]. Except for some electrochemical and optical detecting approaches, for example surface plasmon resonance phenomenon based approaches, the vast majority of the immune-detection strategies are label based. Notably, there are limitations to label based methods that increase cost. Multiple labeled antibodies, labeling the reaction, and immunofluorescence detection equipment increase cost. Therefore, development of label-free immunological techniques where one variant of primary antibody is used would diminish the cost of immunoassays and simplify cancer screening systems.

The cancer biomarker CA125 was discovered in 1981 using the OC125 monoclonal antibody. It is a mucin-like a glycoprotein (MUC16) in nature, and has a molecular weight >200 kDa encoded by the *MUC*16 gene. CA125 is the most extensively studied biomarker in cancer research, presence of high levels of CA125 is the key basis for immunoassays to recognize and screen the progression of epithelial ovarian cancer [38,39]. In addition, further research studies identified the presence of high levels of CA125 in other malignancies such as gastric and breast cancers [40,41,42,43]. The CA125 is a membrane glycoprotein and is soluble in body fluids. Typically, CA125 detecting immunoassays have an identification breaking point of 15 U/mL, which is adequate in view of the 35 U/mL CA125 level corresponding with the infection state [44]. Due to the increased necessity of early diagnosis of cancer, several immunological assays have been reported for the detection of the cancer biomarker CA125 using electrochemical biosensors, nanomaterial-based colorimetric immunoassays, arrayed microsensor chips, bead-based enzyme-linked immunosorbent assay (ELISA), and a fluorescent immunoassay employing the ALYGNSA antibody orientation system [44,45,46,47,48,49,50]. As noted, these label based methods have cost limiting restrictions, but these conventional label based immunoassays can further reduce the detection limit and test volume required. This gives other options for high-throughput screening of cancer biomarkers.

**Figure 4:**
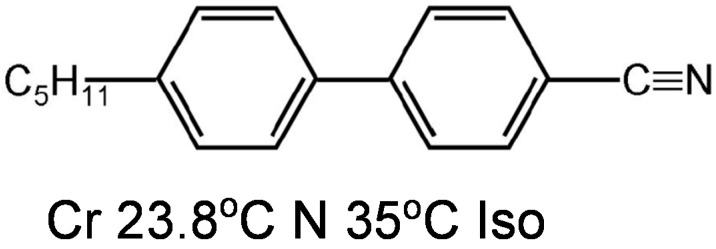
Molecular structure of 4-Cyano-4′-pentylbiphenyl (5CB) and phase transition temperatures (°C) of 5CB. (Cr—Crystal; N—Nematic; Iso—Isotropic).

A group of investigators exploited the use of the liquid crystal 5CB (4-Cyano-4′-pentylbiphenyl, chemical structure shown in Figure 4) as a sensing substance to identify biomolecules. LC-based biosensors have been the center of attention in cancer diagnosis [23,24,25,26,51,52,53,54,55,56,57,58]. The birefringent properties of anisotropic molecules in liquid crystals are exploited in detecting cancer biomarkers. The presence of biomolecules (biomarkers), as well as their binding and reaction nature with other molecules, alters the orientation of LCs and eventually triggers a change in optical appearance [21,53,59]. Currently, LC-based techniques are amplifying the signals of bio-molecules by measuring interaction with each other. Recently, various investigators exploited this property for real-time monitoring of enzymatic reactions [27,28], protein-peptide, protein-protein, and protein-receptor binding [26,52,60,61], immunodetection of specific binding between antibodies and antigens [53,62,63,64,65], recognition of the orientation of immobilized proteins [24,25], glucose bio-sensing [66], and DNA hybridization assays [56,57,67]. The orientation and direction of LC is sensitive to the amount of immunological assay which is applied to the surface. Indeed, when the concentration of biomolecules reach a critical value, the LC generates an optical response from dark to bright within a small concentration range. This characteristic phenomenon change is not observed in conventional methods due to the adsorption of visible or UV light. The optical response produced is very precise and reproducible. This kind of LC based biosensing approach is very beneficial in the detection of biomolecules and increases the potential applications of LCs. In addition, these LCs can be applied for screening of cancer, especially when the readout is a binary negative or positive condition.

In detail, LC involved immunological procedures where the proteins or antibodies are immobilized on a glass substrate and this glass slide is coated with silane surfactants and it is normally dimethyloctadecyl[3-(trimethoxysilys) propyl] ammonium chloride (DMOAP) [60,62,65,68,69]. In the subsequent procedures these proteins and antibodies get adsorbed on the DMO coated layer, however non-specific biomolecules will be get washed and eventually eliminated with the washing procedures and it causes gradual dissociation of the immobilized antibody, antigen, or immunocomplexes. This dissociation will reduce the reproducibility and sensitivity of the immunological assay. Several studies have reported that the binding affinity of the LC aligning agent can increase by modifying the DMOAP layer with the exposure of ultraviolet light (UV) and it leads to enhance the function by providing active functional groups which in turn causes an increase in the binding association between the biomolecules and alignment layer [70,71]. In the above reaction, the UV exposure causes the degradation of functional groups such as -OH, -COOH, and -CHO and the self-assembled monolayer of alkylsiloxane is on the UV-irradiated surface and it will become hydrophilic [72,73,74,75]. The oxidized functional groups which are produced with the help of oxygen by ozonolysis through UV light are essential to interact the biomolecules and cells to reduce the formation of hydrogen and covalent bonds [73,76,77,78]. Therefore, the UV-irradiated DMOAP surface is needed for immobilization of biomolecules. These studies characterize a new method to immobilize biomolecules on a DMOAP surface with good stability. The results describe an experimental approach in preparing and screening capacity of surfaces to oppose the adsorption of biomolecules. These molecular insights, and understanding the mechanisms leading to protein molecules opposing surfaces by associating this peculiar property with molecular structure, are useful when designing new devices.

The sample preparation procedure of LC-based immunological assays has multiple steps [79,80,81]. Initially, the glass slides are cleaned by immersing in detergent solution, deionized water, and finally in 99% ethanol in sequential order and in each step allowing the ultra-sonication for 15 min at room temperature. The cleaned glass slides are dried under the flow of nitrogen and follows baking at 74 °C for 15 min. Later, these glass slides are immersed in 1% (*v*/*v*) DMOAP aqueous solution for 15 min at RT (room temperature). The excess DMOAP from these glass slides is removed by ultra-sonication in deionized water for 1 min and allowed for drying by using the flow of nitrogen and heating at the 100 °C for 15 min. In the next step, UV modified DMOAP layer glass slides are prepared by irradiating the DMOAP coated glass slide with UV light at 15 mW/cm^2^ for 0–20 min. Afterwards the CA125 antibody is immobilized on these UV modified DMOAP coated glass slides. The CA125 antibody immobilized DMOAP coated glass slide is dried at room temperature overnight, and the next day the CA125 antigen is dropped in a desired concentration of the immobilized antibody DMOAP coated glass slide, and follows the exposure to the substrate with a clean cover glass to form a cell. This allows the interaction of the CA125 antibody and antigen and it should be carried out at room temperature for 2–3 h. In the following step, the cover glass is removed and rinsed with deionized water and afterwards dried by using a hot plate to remove excess water. The LC cell is prepared for optical studies by pairing two DMOAP coated glass slides, one without any further treatment and one with an analyst immobilized in an array format. In the final step, LC is filled through capillary mechanism. The LC cell optical texture is observed under a microscope in the transmitted light illumination by using crossed polarizers [79,80,81]. The fundamental mechanism behind this principle is a change in the optical texture of LCs, directly proportional to the number and type of biomolecules from the analyst source present in an LC aligning layer.

The quantitative type of LC-based immunological assay has been developed that correlates antibody concentration with the length of a bright region in the LC optical texture [65]. This bright region results from the interaction of antibodies with immobilized antigens. Xue et al. proposed that the optical texture of LCs is related to the concentration of antibodies or proteins, and that there exists a critical concentration which can induce a dark to bright response for each individual antibody or protein [53]. A study on an LC-based immunoassay of hepatitis B revealed that the immunocomplex formed between hepatitis B surface antigen (HBsAg) and hepatitis B antibody (anti-HBsAg) interrupts the homeotropic alignment of LC, returning to the planar texture as observed under the polarizing microscope. In addition, the detection limit of anti-HBsAg drops from 150 nM to 15 nM when a secondary antibody specific for anti-HBsAg is added [62]. This increases the size of the immunocomplex and thus creates greater disturbance in LC orientation.

Su et al. (2015) [81] proposed a method to enhance the sensitivity of LC-based immunodetection of the cancer biomarker CA125 without the use of secondary antibodies. They achieved this by exploiting a eutectic mesogenic mixture exhibiting large anisotropy in refractive index such as *n*_o_ 0.333 at 20 °C. Additionally, the authors have demonstrated that the sensitivity of LC-based immunoassays can be promoted by applying a eutectic mixture with larger birefringence. This study shows that the cancer biomarker CA125 detection region is consistently 1–500 ng/mL which is sufficient for clinical diagnosis. The presence of specific antibodies and formation of immunocomplexes can be clearly distinguished from nonspecific antibodies through inspection of LC optical textures.

In 2014, Su et al. developed an optical LC immunodetection technique based on the use of a label-free array. They suggested that LCs with larger birefringence are more sensitive in detecting biomolecules compared with conventional LCs. Additionally, they pointed out that choosing an appropriate antibody concentration in the design of the antibody array is crucial in the sensitivity of LC-based immunodetection. Higher concentration of the cancer biomarker CA125 resulted in greater disruption of the optical texture. A similar disruption is not seen when the anti-CA125 antibody is replaced with nonspecific antibodies. LC-based immunodetection is specific and the change in its optical texture can be correlated with the analyte concentration. The detection limit of LC-based immunodetection for CA125 is within 0.01 to 0.1 µg/mL, significantly lower than the 4 to 5 µg/mL of fluorescence immunoassay [80]. The detection limit for the cancer biomarker CA125 can be further improved with UV modified DMOAP alignment layers. A simple UV modification method is proposed, by Su et al. (2014), to increase oxygen functional groups on the DMOAP alignment layer thus enhancing binding affinity and efficiency towards anti-CA125 antibodies. Compared to an unmodified counterpart, immunodetection on UV modified DMOAP alignment layers exhibited considerably lower detection limits and more significant changes in LC optical texture in the presence of the CA125 antigen [80]. Therefore, the detection limit of LC-based immunoassay techniques is improved by surface modification of the DMOAP surfactant alignment layer. As shown in this study, the cancer biomarker CA125 is detected with antibodies immobilized on the UV modified a DMOAP surfactant layer. When compared to an untreated sample, the UV irradiated sample enhanced the binding affinity and reproducibility of the CA125 antibody assay.

Therefore, these results suggest that LCs with a large birefringence are more sensitive in detecting biomolecules compared with the conventional LCs. It also indicates that modification of the alignment layer by UV radiation enhances the performance of the label-free LC-based immunodetection. This makes label-free LC-based immunodetection a viable alternative method to conventional label based methods. Additionally, choosing an appropriate antibody concentration is critical in determining the sensitivity of LC-based immunodetection, and therefore, there is still room for improvement of these techniques. In summary, LC-based immunoassays hold great promise for the development of cancer screening technologies and have great potential to replace conventional immunoassays methods.

### 2.2. Detection of Keratin Forming Cell Tumor Cell Line Type B (KB) Cancer Cells: Label-Free In Vitro Approach

The configurational behavior of LC microdroplets emulsion makes them attractive for the detection of interfacial interactions of chemicals or biomolecules in biological systems [28,53,82,83,84,85]. Configurational transitions in LC emulsions are detected using an optical microscope under crossed polarizers, regardless of species or stimuli. The LC detection system for KB cancer cells uses 5CB liquid crystal emulsions prepared using folic acid conjugated block copolymer in the presence of sodium dodecyl sulfate, which acts as a mediator of configurational transitions [86].

**Figure 5:**
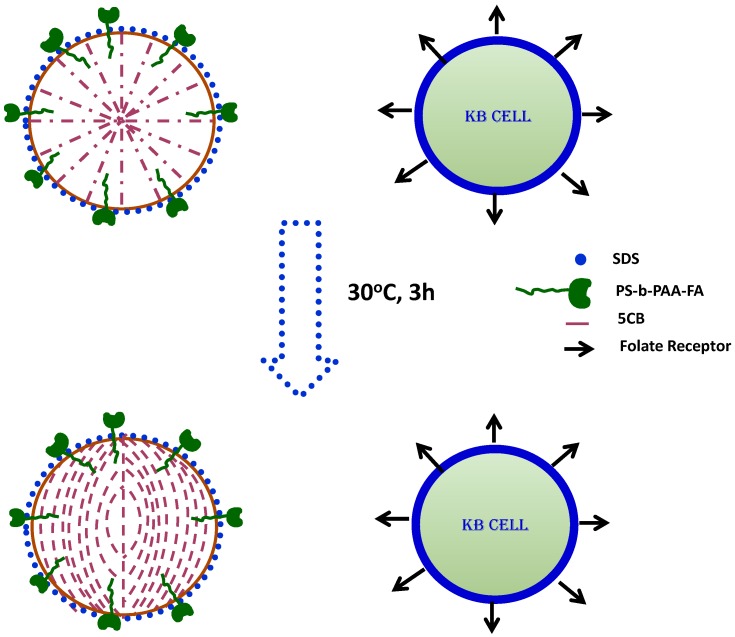
Detection of KB cancer cells by liquid crystal (LC) microdroplets emulsion: KB cancer cells interact with LC microdroplets. These LC microdroplets contain folic acid (conjugated block) that allows the radial configuration formation through co-polymerization.

To prepare the LC microdroplet emulsion, a calculated amount of folic acid conjugated block copolymers is dispersed in a solution of phosphate-buffered saline (PBS) and then sodium dodecyl sulfate (SDS) is added. The LC emulsion is prepared by a dropwise addition of 5CB in an aqueous solution of block copolymers and SDS. Subsequently, the LC microdroplet emulsion was used to incubate with KB cancer cells. The LC configurational state is then evaluated by optical behavior. Free liquid crystal microdroplets showed radial configuration whereas liquid crystal microdroplets in contact with KB cancer cells showed a configurational transition from radial to bipolar (Figure 3). The interaction of KB cancer cells with the LC microdroplets was strong and specific due to biological interactions of KB cancer cells folate receptors with folic acid ligands anchored on the surface of LC microdroplets. These interactions were so robust that the same configurational transitions were found in detection of virus and bacteria (Figure 5) [30,87,88,89,90,91]. Importantly, the interaction of folic acid anchored LC microdroplets with KB cancer cells folate receptor is not only sensitive but also selective for the detection of KB cancer cells.

These findings suggest that this system could be used to develop a biosensor for the label-free detection of KB cancer cells in biological systems and that it is also potentially useful for in vitro detection of KB cancer cells.

### 2.3. Early Detection of Skin Cancer: By Using a Liquid Crystal Associated Device

The skin is the outer covering of the body that serves many functions including sensation and temperature regulation. Additionally, it protects the bones, muscles, ligaments, and internal organs from pathogen exposure, chemical insults, and UV radiation. Damage to the skin can occur through both external and internal factors including burns, injuries, and tumors. Damage is repaired through wound healing which functions through epithelialization of preserved dermis or formation of a scar. Inflammatory response and regenerative processes make the border between healthy and damaged tissues difficult to define. This determination is critical for clinical diagnosis of skin cancer and treatment of moles, tumors, and other cutaneous lesions. At early stages, skin cancer is largely curable but after metastasis occurs it becomes resistant to most current medical treatments. Typically, skin cancers start as harmless pigmented lesions which makes them hard to diagnose but develop and result in disastrous metastasis. This poor outcome makes clear the necessity of early detection of skin cancer. The method most commonly follow by physicians for diagnosis of skin cancer is the ABCDE rule. The ABCDE outlines characteristics for physicians to investigate as follows: Asymmetric shape (A), border (B), color (C), diameter (D), and evolution (E) [92]. Performance of dermatosis examination continues to be influenced by physician experience leading to elevated rates of false positive skin cancer diagnoses. However, for the last 35 years, researchers have endeavored to create noninvasive, simple, and time-efficient methods to diagnose skin cancer. Among them, methods based on light scattering [93,94], confocal microscopy [95], optical coherence tomography (OCT) [96,97,98], photo-acoustic microscopy [99], polarimetric imaging [100,101,102], and spectropolarimetric imaging (SP) [103,104,105]. We can investigate skin lesions from the microscopic and macroscopic behavioral changes. Morphological changes that are associated with precancerous disease will alter the measurement of many factors including: Tissue absorption and scattering of light as well as other fluorescent characteristics of the skin. For example, collagen denaturalization will affect birefringence as a result of distortion of typical molecular binding structure [106,107,108]. Therefore, polarized optical microscopy and spectroscopy can be used to test for precancerous signals. Recently, there has been increased interest in macroscopic scale diagnosis. This is based on changes in epithelial architecture of early stage cancer cells before they have become invasive where changes alter scattering and other optical parameters [101,109,110]. This phenomenon was initially observed and characterized extensively in skin tissues where changes in the polarization state of backscattered light from a turbid medium occurred [111,112]. This method was successfully used for skin lesion boundary detection in Mohs micrographic surgery (MMS) [103,113]. In addition, Boulesteix et al. developed a method for stained hepatic biopsies that determines the degree of polarization at visible and near-infrared spectral regions using Muller matrices [114]. This allowed Boulesteix et al. to emphasize the anomalous structure of collagen at different wavelengths. Around the same time of the Boulesteix method, Ramella et al. created a method using two charge coupled device (CCD) cameras which simplified the polarization readout from the tissue and allowed them to calculate the normalized contrast between the cameras simultaneously [102]. Weber et al. built upon these methods by altering the cross and parallel polarizations separately enhancing resolution and increasing diagnostic capabilities [115]. Ramella et al. in 2005, once again improved upon previous methods. Here, Ramella et al. used skewed illumination for backscatter imaging which eliminated glare with no need for refractive index-matching materials such as oil or water, and thus greatly increased the ability to illuminate tissue [116].

**Figure 6:**
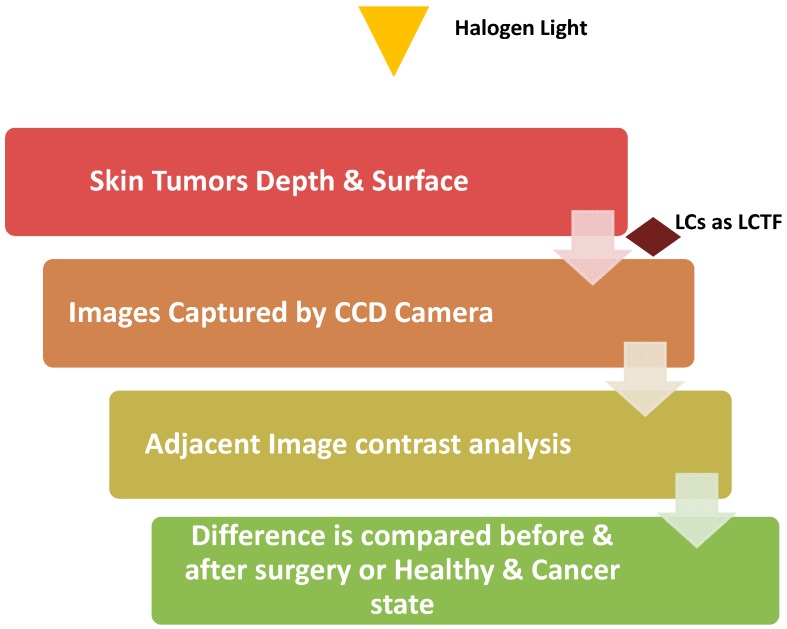
Schematic representation of spectropolarimetric imaging system (DOSI).

In 2011, Aharon et al. designed a prototype based on their hypothesis that changes resulting from disease will create changes in the backscattered light and that these changes can help diagnosis of precursors to cancer. Non-invasive detection systems such as differential optical spectropolarimetric imaging (DOSI) system distinguishes intradermal structural changes in precancerous tissue using two wavelengths to allow continuous polarimetric control. The principle behind DOSI is to collect the information from the surface as well as from within skin tumors using of liquid crystal devices. The DOSI imaging system using LCs is shown in Figure 6, and it comprises a halogen light source with collimated and imaging optics of liquid crystal tunable filter (LCTF) [117,118] with linearly polarized output, and also a wavelength independent liquid crystal polarization rotator (WILCPR) [119,120]. A CCD camera with fixed polarizer captures a sequence of images. Analysis is performed using computer programs that measure contrast and average the chosen section of two images. Since collagen is not well organized and the fibrils texture orientation of collagen depends on its location in the skin, the DOSI imaging system was designed to bypass these issues. As Aharon et al. described, this system can “scan bipolarization states by continuously rotating the linearly polarized light incident on a skin lesion and collecting differential contrasts between sequenced images, while simultaneously averaging the statistical readout of a (CCD) video camera” [117]. This differential spectropolarimetric skin imaging system uses the advantage of liquid crystal devices, either a polarization rotator or a tunable filter which operate at a spectral range from visible to near-infrared. The method is non-invasive as it predicted tumor location by assessing the skin for areas of statistical polarization change indicated by a change is the scattering of light from the lesion. This technique can supplement epiluminescence microscopy. In vivo imaging of skin tumors using this method before patients underwent surgery compared to histopathological results indicate that this differential spectropolarimetric skin imaging system offers a noninvasive approach as a new way to tackle precancer diagnoses.

## 3. Liquid Crystal Chemotherapeutic Drugs for the Treatment of Cancer (LCs as New Antitumor Drugs)

There are currently three major therapy categories for cancer treatment being (1) surgery, (2) radiation therapy, and (3) chemotherapy. Chemotherapy uses various drugs that regulate the growth of abnormal cancer cells. Chemotherapy is frequently combined with surgery or radiation therapy. Most chemotherapeutic drugs are anthracycline, plant alkaloids, antimetabolites, monoclonal antibodies, topoisomerase inhibitors, or other antitumor agents [4,5,6,7,121,122,123,124,125,126]. Even though various types of chemotherapeutic drugs have been developed recently, only very few drugs have led to complete recovery in cancer patients [9,127,128]. Therefore, development of chemotherapeutic drugs is essential for worldwide treatment of cancer patients.

**Figure 7:**
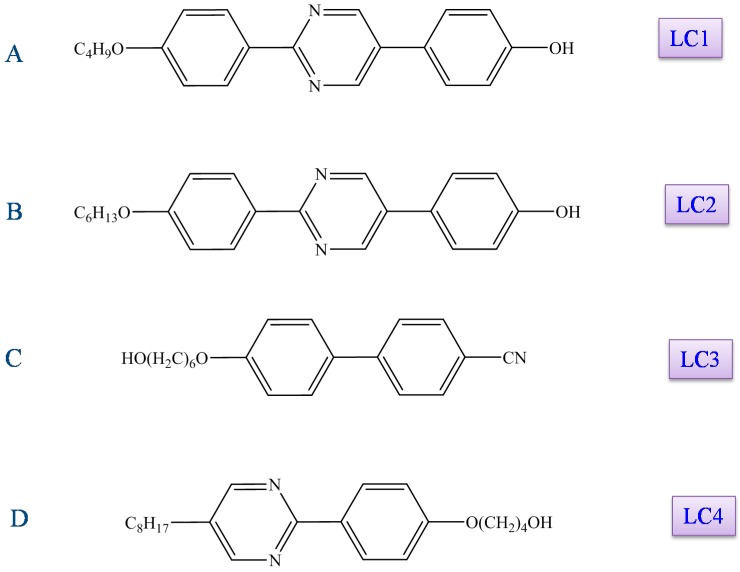
Structures of different types of liquid crystals.

Many studies have explored LCs for novel anti-cancer functions. One of the main interests has been the biological and pharmacological activity of amphiphilic liquid crystalline compounds including: d-glucamine possessing phenylpyrimidine derivatives and cyanobiphenyl derivatives with a terminal hydroxyl unit (Figure 7). Following this interest, four compounds were evaluated for their anti-cancer function: 2-(4-butoxyphenyl)-5-(4-hydroxyphenyl)pyrimidine (LC1), 2-{4-(4-hexyloxyphenyl)phenyl}-5-hydroxypyrimidine (LC2), 4′-[(6-hydroxyhexyl)oxy]-[1,1′-biphenyl]-4-carbonitrile (LC3), and 4-(4-(5-octylpyridin-2-yl)phenoxyl)butan-1-ol (LC4). These LCs showed marked cell growth suppression, cell cycle arrest at the G2/M phase, and apoptosis [121,129,130,131,132,133,134]. Chronic myelogenous leukemia K562 cells, A549 human lung cancer cells, and U937 human leukemic monocyte lymphoma cells were used to test LCs anti-cancer properties by evaluating cell growth, cell cycle distribution, and signaling pathways using flow cytometry and Western blot analysis.

## 4. LC1 and LC2 Suppressive Effects on K562 Cell Growth

Chronic myelogenous leukemia (CML) is an unusual type of cancer which affects bone cells and bone marrow, and this hematopoietic stem cell disease is signified by presence of the Philadelphia chromosome (abnormality in chromosome 22 and seen in leukemia, i.e., CML). Translocation of the Philadelphia chromosome converts a chimeric protein Bcr-Abl tyrosine kinase. BCR-ABL dependent kinase activity regulates multiple crucial pathways related to cancer growth including cell proliferation, differentiation, migration, adhesion, and apoptosis [135,136,137,138]. Molecular targeting of BCR-ABL using an inhibitor, STI571, has shown promise in treatment of CML patients based on cytogenetic remission, but has been poor for later stage treatment with most cases resulting in relapse [137,139,140]. Although this is disappointing, these outcomes are not unique to leukemia patients. Over time, almost all tumors eventually develop resistance to chemotherapeutic agents, and it is this resistance that makes it crucial to develop new, more effective drugs and treatments [141].

Both LC1 and LC2 showed cell growth suppression in K562 cells at a treatment in the µM range. The suppressive effects can be related to the specific chemical structures of LC1 and LC2 [133]. LC1 and LC2 both consist of a core of three aromatic rings (pyrimidine ring with one alkyl chain and one hydroxide on either side). A rigid position of aromatic rings conveyed the suppressive effect. LC1 induces apoptosis while LC2 does not, despite sharing a powerful suppressive effect on growth in K562 cells at equivalent doses. There are structural differences between the two compounds that likely confer the differences in function including: the length of the alkyl chain and position of the pyrimidine rings. LC1 appears to act by initiating programed cell death resulting in a decrease of survival signal and the increase of death signal. It is possible that LC1 may act through JNK, p38, and Erk. JNK and p38 are activated though multiple extracellular cues that induces the stress response and apoptosis, while Erk regulates cell survival and proliferation [142,143]. On the other hand, LC1 could also act through Bcl-xL. In K562 cells, apoptosis is regulated through reduced expression Bcl-xL, an anti-apoptotic protein [130,144,145]. Yukako Fukushi et al. (2011) showed that LC1 treatment reduced expression of Bcl-xL [133]. LC1 treatment and Bcl-xL reduction may lead to apoptosis through release of cytochrome from mitochondria. Induction of apoptosis can also occur via reactive oxygen species (ROS), and this has also been shown in K562 cells [3,122,123]. ROS can act as a stress trigger or a second messenger of intracellular signal transduction. In fact, in K562 cells treated with LC compounds, ROS induction was detected and it is possible that this ROS production is the trigger for apoptosis after LC1 treatments [133,146,147,148]. Further experiments are needed to determine if ROS production by LCs is necessary to directly or indirectly signal death. Interestingly, only LC1 induced activation of p38 mitogen-activated protein kinase, c-Jun N-terminal kinase, and apoptosis of K562 cells, while LC2 did not activate these signaling pathways and ultimately did not alter cell cycle distribution. Even though the molecular mechanism of action for LC2 remains unclear, the contrasting results between LC1 and LC2 indicate that method of cell proliferation suppression is likely different.

## 5. Apoptosis and Cell Cycle Arrest by LC1 and LC2

In 2011, Takahashi et al. reported the biological and pharmacological potential of some of the liquid crystalline compounds including cyanobiphenyl derivatives (terminal hydroxyl) and phenylpyrimidine derivatives (containing d-glucamine). They explored anti-cancer properties of liquid crystals, especially LC3 and LC4 in A549, human non-small cell lung cancer, cells and both showed suppressed cell growth through G1 phase arrest but not cell death [134]. A549 cells treated with liquid crystals (10–30 µM for 24 h) showed a correlation in flowcytometry experiments between cytostatic activity and G1 phase arrest. However, in WI-38, normal fibroblast cells, LC3 did not induce any suppression or G1 phase arrest even though in A549 cells it showed high growth suppression. On the other hand, in A549 cells, LC4 induced G1 phase arrest but also attenuated the growth of WI-38 cells. Neither LC3 nor LC4 induced cell death. In contrast, Takuya et al. (2013) showed that liquid crystal compounds with three aromatic rings (LC1 and LC2) increased potential to induce apoptosis [149]. In A549 and WI-38 cells treated with LC1 and LC2 (6–12 µM) showed 95% and 78% inhibition respectively. In A549 cells, LC3 and LC4 treatment inhibited cell growth through G1-phase arrest without any induction of cell death. However, LC1 and LC2 increased sub-G1 fraction and Annexin V-positive cells as well as activating caspase-3. These results indicate LC1 can induce apoptosis through a caspase-dependent pathway. Interestingly, treatment with a caspase inhibitor did not rescue suppression of cells by LC1. It is possible that this occurred due to the specific inhibitor used, in that it may not have prevented cell cycle arrest. Fukushi et al. reported that in K562 cells (CML cell line), LC1 activated JNK (c-Jun N terminal Kinase) [133]. In contrast, JNK inhibitor did not affect A549 cell suppression by LC1. Interestingly, the effect of LC1 did not inhibit the proliferation of WI-38 cells. It reveals LC1 and LC2 have different mechanisms for inducing growth suppression of A549 cells.

LC1 shows promise for development of new chemotherapeutic drugs due to the suppressive effects observed. However, we need to understand the mechanisms of cell growth suppression by different liquid crystalline compounds. Even though the exact mechanisms are unclear, perhaps the chemical structure is responsible for different anti-cancer behaviors. Therefore, future studies will be crucial for uncovering the molecular mechanisms and structural relationships among LCs that will allow for development of more powerful and tumor-specific drugs.

## 6. Toxic Properties of Liquid Crystals on the Growth of Cells

Until now, it was not clear whether liquid crystals have any cytotoxic properties against leukemia cells which are normally treated by chemotherapeutic approaches. To probe this question, Ishikawa et al. studied the cytotoxic properties of liquid crystals against the human leukemic monocyte lymphoma cell line U937 [147]. Among the LCs (Figure 4), LC3 and LC4 shows marked cytotoxicity. However, the cytotoxic effects of these liquid crystals have no relation to induction of apoptosis. The cytotoxic property of an LC depends on the cell type as the liquid crystal compounds which are cytotoxic to U937 cells may not be cytotoxic to other cell types. For example, LC3 and LC4 have little effect on A549 cells. Therefore, it would seem that some property underlies the cell-specific cytotoxic effects and it seems likely the structure of liquid crystals might be responsible. A specific example supporting structure dictating the effect can be found with LC4. LC4 did not exert cytotoxic effects against A549 cells but showed a strong cytotoxic effect in U937 cells. The results of further analyses of LC4 indicated that the molecular structure of liquid crystals was responsible for the cell associated cytotoxic nature [147]. The liquid crystals which show cytotoxic nature with U937 cells are due to rod-like molecules possessing -O(CH_2_)_6_OH in their liquid crystal structure. These LCs contain a flexible spacer containing an alkyl chain but are not associated to a large polar group such as -CN, -OH or -COOH through this versatile spacer. Even though it seems probable that the kind of structure could be responsible for the cytotoxic nature of some LCs, further investigations need to be done to greater understand the relationship between liquid crystal structure and its activity.

## 7. Conclusions and Future Perspectives

This review article summarizes the recent developments in the discovery of cancer biomarkers such as nucleic acids, proteins, and enzymes with an emphasis on LCs and application of LCs for development of novel drugs for both detection and treatment of cancer. We discussed how multidisciplinary technology based cancer diagnostics are beginning to replace traditional techniques. Even though much more work is needed to translate these multidisciplinary technologies into viable cancer diagnostics and treatment options, the exploration of new LC based technologies for advanced cancer diagnostics is gaining interest invariably. Even though, enormous advancement has been made over the past decades in identifying cancer biomarkers, assay creation, and in the generation of new drugs for cancer therapy, most of the advances are only preliminary work demonstrating potential application and not advanced work leading to clinically viable applications. This is frequently due to assays being optimized to conditions in the laboratory that do not extend into the clinical environment. There are some challenges which remain as obstacles before these technologies can be translated from a research environment to clinical application. Primarily the requirements for optimal LC performance, notably structure-activity relationships and the molecular mechanisms of LCs, have not been studied exhaustively. First, the performance of LC-based bio-sensing depends on its selectivity, sensitivity, detection limit, reproducibility, response time, cost, and temporal resolution. However, for cancer diagnosis the biosensor should have the sensitivity to transduce identification events to a readout signal which is essential. Therefore, ceaseless endeavors have been devoted to enhance existing strategies or to discover elective procedures for the quantification of cancer biomarkers. Furthermore, how the techniques’ reliability and robustness intensify the signal is crucial in the application of these methods for tracking cancer biomarkers from a clinical point of view. To date, the research on the liquid crystal based immunological assays shows promising results for the advancement of cancer screening techniques, and, additionally, has demonstrated potential to replace traditional immunological detection methods. Undoubtedly, in the near future, LC-based immunoassays will undergo additional developments and refinements to meet clinical diagnostic requirements. Second, some types of LCs show an ability to suppress cell growth. Despite this, LCs’ extraordinary capacity to reduce the cell growth being shown, the mechanism behind the suppression of cell growth suppression, is not clear. Therefore, detailed investigations about LC molecular mechanisms and structure-activity response is required for the advancement of tumor-specific and high potential chemotherapeutic liquid crystal based drug therapies.

With great demand for improvements in clinical diagnostics, modern medicine, and development of cancer biomarker detection and treatment, LC-based immunological assays will likely be of great and growing interest. Future studies will aim to develop high-throughput assays with increased sensitivity and selectivity, miniaturization, and versatility while continuing to identify novel biomarkers for early cancer diagnosis. Further advancement and use of liquid crystals in cancer biology and modern medicine will likely be achieved through multidisciplinary collaborations among chemists, biologists, physicists, clinicians, material scientists, and engineers.

## Abbreviations

5CB	4′-*n*-pentyl-4-cyanobiphenyl
ATM	Ataxia telangiectasia mutated
CML	Chronic myeloid leukemia
DI	Deionized
DMOAP	Dimethyloctadecyl[3-(trimethoxysilys)propyl]ammonium chloride
DOSI	Differential optical spectropolarimetric imaging system
HBsAg	Hepatitis B surface antigen
LC	Liquid crystal
LCTF	Liquid crystal tunable filter
LCRC	Liquid crystal related compound
MMS	Mohs micrographic surgery
NSCLC	Non-small cell lung cancer
OCT	Optical coherence tomography (OCT)
PBS	Phosphate-buffered saline
POM	Polarized optical microscope
ROS	Reactive oxygen species
SP	Spectropolarimetric imaging
UV	Ultraviolet
WILCPR	Wavelength independent liquid crystal polarization rotator
